# Prospective associations between internet use and poor mental health: A population-based study

**DOI:** 10.1371/journal.pone.0235889

**Published:** 2020-07-23

**Authors:** Becky Mars, David Gunnell, Lucy Biddle, Judi Kidger, Paul Moran, Lizzy Winstone, Jon Heron

**Affiliations:** 1 Population Health Sciences University of Bristol, Bristol, United Kingdom; 2 NIHR Biomedical Research Centre at the University Hospitals Bristol NHS Foundation Trust and the University of Bristol, Bristol, United Kingdom; National Institue on Drug Abuse, UNITED STATES

## Abstract

**Objectives:**

Most of the evidence on the effects of internet use on mental health derives from cross-sectional research. We set out to explore prospective associations between internet use (hours online and specific internet experiences) and future mental health problems.

**Methods:**

Participants were 1,431 respondents from the Avon Longitudinal Study of Parents and Children (ALSPAC), a UK birth cohort, who completed a questionnaire on internet use (time online and ten different internet experiences) when they were aged 18 years. Outcomes included past year self-harm, assessed at 21 years and high levels of depression and anxiety symptoms, assessed at 22 years. Associations were investigated using logistic regression models and analyses were conducted separately for males and females.

**Results:**

Females reporting high levels of internet use (number of hours online) were found to be at increased risk of depression at follow-up (highest tertile vs lowest tertile OR = 1.41, 95% CI 0.90 to 2.20), whereas males with high levels of internet use were at increased risk for self-harm (highest tertile vs lowest tertile OR = 2.53, 95%CI 0.93 to 6.90). There was no evidence to suggest an association between hours spent online and anxiety. With regards to the specific internet experiences, associations were found for females but not for males. In fully adjusted models, being bullied online (OR = 1.76, 95% CI 1.09 to 2.86) and meeting someone face to face (OR = 1.55, 95% CI 1.00 to 2.41) were associated with an increased risk of future depression. Being bullied online was also associated with an increased risk of future self-harm (OR = 2.42, 95% CI 1.41 to 4.15), along with receiving unwanted sexual comments or material, and coming across pornography and violent/gruesome material.

**Conclusions:**

Our findings highlight the importance of digital citizenship training to help teach young people to use technology safely and responsibly.

## Introduction

The internet provides opportunities for communication, education, and entertainment and has become an integral part of modern life. The use of the internet has grown exponentially worldwide, particularly among younger age groups,[1] and this rise has led to concerns over potential negative effects. For example, the rise in popularity of social media has been posited as a potential explanation for recent increases in mental health problems among adolescents.[2, 3] There are also concerns about online experiences such as cyberbullying, which has been linked to a range of adverse mental health outcomes.[4–7]

A growing number of studies have found an association between high levels of internet use in young people and poor mental health, including depression, anxiety, attention deficit hyperactivity disorder, hostility/aggression, suicidal ideation, and self-harm.[3, 8–16] However, most existing research is based on cross-sectional data and so the temporal relationship between mental health problems and internet use is currently unclear. The lack of longitudinal studies assessing the consequences of screen-based activities was recently highlighted as a key limitation in the field.[17] Further research is also needed to understand the role of gender. Internet addition is more prevalent in males[18] and there are gender differences in patterns of use, with males spending more time on online games and females on social networking.[19] Research exploring gender differences in association with mental health outcomes is limited but some studies have suggested that findings may be stronger for females.[20, 21]

It is likely that the nature of online experiences, in addition to time spent online, may be important. With the exception of cyberbullying, which has been the focus of multiple systematic reviews,[17] relatively little is known about the relationship between specific online experiences (such as exposure to sexual or violent content) and later mental health outcomes. The existence of prospective associations between online experiences and poor mental health would have important implications for policy makers, clinicians, parents and teachers, as well as the providers of internet services. The current study uses data from a population-based birth cohort to investigate the following research questions:

1. Is there an association between time spent online at age 18 years and mental health problems (depression, anxiety and self-harm) at 21/22 years?
2. Are there prospective associations between specific internet experiences (e.g. being bullied online, exposure to unwanted sexual content) and later mental health problems?
3. Do associations differ for males and females?

## Methods

### Sample

The Avon Longitudinal Study of Parents and Children (ALSPAC) is an ongoing population-based birth cohort study examining influences on health and development across the life-course. The ALSPAC core enrolled sample consists of 14,541 pregnant women resident in the former county of Avon in South West England (UK), with expected delivery dates between 1^st^ April 1991 and 31^st^ December 1992.[22–24] Of the 14,062 live births, 13,798 were singletons/first-born of twins and were alive at one year of age. Participants have been followed-up regularly since recruitment through questionnaires and research clinics. The study website contains details of all the data that is available through a fully searchable data dictionary http://www.bristol.ac.uk/alspac/researchers/our-data/. Ethical approval for the study was obtained from the ALSPAC Ethics and Law Committee and the Local Research Ethics Committees. Informed consent for the use of data collected via questionnaires and clinics was obtained from participants following the recommendations of the ALSPAC Ethics and Law Committee at the time.

**Fig 1:**
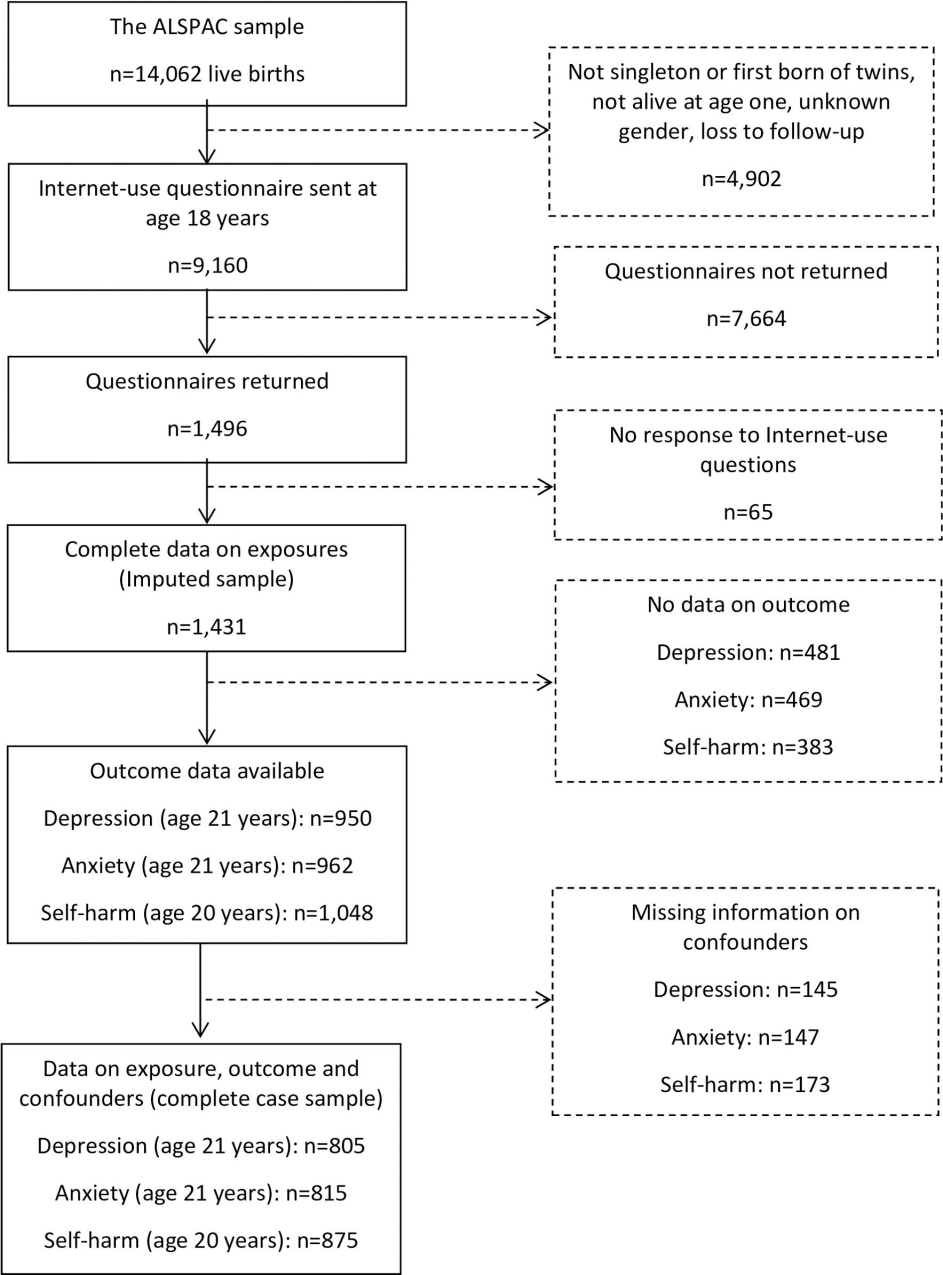
Flow-chart of attrition in the Avon Longitudinal Study of Parents and Children (ALSPAC) birth cohort.

In the present investigation, we investigate later mental health outcomes amongst 1,496 participants who completed a detailed online self-report questionnaire on internet use in 2010, when they were 18 years old (mean age of respondents 18 years 2 months, standard deviation (SD) 6 months). The proportion of the sample who completed the questionnaire was 16.3% (1,496/9,160). Complete data on exposures of interest (hours online and internet experiences) was available for 1,431 individuals (See Fig 1 for a flowchart of data availability). Questionnaire responders were more likely than non-responders to be female, have a higher parental social class and have a mother with higher education and a lower parity; they were also less likely to have experienced over-crowding (S1 Table). Outcomes were assessed via self-report questionnaire at age 21 years (for self-harm) and 22 years (for depression/anxiety symptoms). Participants could choose to complete these via post or online. Outcome data were available for n = 1,048 for self-harm, n = 950 for depression symptoms and n = 962 for anxiety symptoms.

#### Data access

This study involved analysis of existing data from the Avon Longitudinal Study of Parents and Children (ALSPAC) birth cohort. The data were accessed in line with ALSPAC’s access policy, which is available on the study website http://www.bristol.ac.uk/alspac/researchers/access/

### Measures

#### Exposures

*Time spent online*. Participants were asked how many hours they spent online each week. The total number of hours was then categorised into a 3-level categorical variable based on the distribution of scores (tertiles) to represent low, moderate, and high levels of internet use. This variable was derived separately for males and females to allow for gender differences in use.

*Internet use experiences*. Young people were asked whether they had ever experienced each of ten different scenarios when using the internet and the frequency with which these occurred (coded from 1 (never) to 5 (always)). These included visiting unmoderated chatrooms; being bullied online; receiving unwanted sexual comments; coming across pornography; being sent unsolicited sexual material; coming across violent or gruesome images; coming across racist or hateful material; meeting someone face to face that they first met online; having personal information they disclosed misused by another person and receiving junk mail/unwanted email. Items were recoded into binary variables to reflect whether the scenario was experienced or not (never experienced vs ever experienced).

#### Outcomes

*Depression symptoms*. Depression symptoms were assessed at age 22 years (mean age of respondents 21.8 years) using the short mood and feelings questionnaire (sMFQ). The sMFQ is a 13-item self-report questionnaire used to measure the severity of depressive symptoms in the previous two weeks.[25] Scores range from 0 to 26 with higher scores indicating more severe depressive symptoms. The sMFQ scale was dichotomised, with scores of 11 indicating high risk of depression. This cut-point has been applied in previous studies,[26–28] and has shown high discriminatory ability against International Classification of Diseases (ICD)-10 diagnostic criteria for depression in early adulthood.[29]

*Anxiety symptoms*. Anxiety symptoms were assessed at age 22 years (mean age of respondents 21.8 years) using the Generalised Anxiety Disorder Assessment (GAD-7). The GAD-7 is a seven item self-report questionnaire used to measure the severity of symptoms of generalised anxiety disorder in the previous two weeks.[30] Scores range from 0 to 21 with higher scores indicating high risk of an anxiety disorder. The GAD-7 was dichotomised, with scores of 10 or more indicating high levels of anxiety symptoms. This cut-point has been shown to have high sensitivity and specificity for detecting GAD.[30–32]

*Self-harm*. Self-harm was assessed at age 21 years (mean age of respondents 20.8 years) with the question: “Have you ever hurt yourself on purpose in any way (e.g. by taking an overdose of pills or by cutting yourself)?” Participants who responded positively were then asked a follow-up question to determine the last time they self-harmed (responses: in the last week, more than a week ago but in the last year, more than a year ago). This item was recoded into a binary variable to indicate whether the young person had self-harmed in the previous year.

*Possible confounders*. Analyses controlled for the possible confounding effects of socioeconomic position (SEP) and previous mental health problems. SEP was assessed via maternal questionnaire and included i) highest of maternal/paternal social class, assessed during pregnancy (professional/managerial or other), and ii) highest maternal educational attainment, assessed during pregnancy (less than O-level, O-level, A-Level or university degree). Previous mental health problems were assessed at age 16 years using the sMFQ. In addition, when exploring associations with each of the internet use experiences, we also adjusted for the total numbers of hours spent online, as we anticipated that individuals who spent more time online would be more likely to encounter potentially harmful web-based exposures.

### Analysis

Multiple logistic regression models were used to explore associations between measures of internet use (hours online and the ten different internet experiences) and three mental health outcomes (depression, anxiety and self-harm). With regards to the internet experiences, we included receiving junk mail/unwanted email as a negative control, as we felt it was unlikely to influence later mental health. The inclusion of negative controls such as this can help to detect potential sources of bias in observational studies. Secondary analysis explored associations between the total number of internet experiences (range 0–10) and mental health outcomes. Analyses were conducted separately according to gender and conducted using Stata version 15.

#### Missing data

Our analyses were conducted on an imputed dataset based on 1,431 individuals with complete exposure data (data on hours online and internet experiences) at age 18 years. Multiple imputation by chained equations [33] was used to generate 20 imputed datasets in which missing outcome and confounder data are replaced by imputed values sampled from their predictive distribution. Multiple Imputation assumes that data are missing at random (MAR), whereby any systematic differences between the missing and the observed values can be explained by differences in observed data. Findings were broadly consistent across the complete case and imputed samples (S2 and S3 Tables).

## Results

Twenty percent of the sample were at high risk of depression at follow-up (22% females; 16% males); 12% were at high risk of an anxiety disorder (14% females; 9% males) and 12% reported having self-harmed in the previous year (13% females; 10% males).

### Time spent online

The median number of hours spent online per week was 14 (Interquartile range (IQR) 7 to 21). Males spent more time online than females (median for males = 15, IQR 10 to 26.5; median for females = 10, IQR 7 to 20). When modelled as a continuous variable, total number of hours online was associated with an increased risk of depression (fully adjusted OR per hour increase = 1.01, 95% CI 1.00 to 1.02, P = 0.047) and self-harm (OR = 1.01, 95%CI 1.00 to 1.03, P = 0.056), but not anxiety (OR = 1.00, 95%CI 0.99 to 1.02, P = 0.310). We did not find statistical evidence to suggest a departure from a linear relationship (depression P value = 0.642; anxiety P value = 0.477; self-harm P value = 0.615).

When compared with low levels of internet use (lowest tertile) females in the highest tertile had an increased odds of depression (unadjusted OR = 1.60, 95% CI = 1.06 to 2.44) (Table 1). Findings remained consistent following adjustment for SEP and previous mental health problems (fully adjusted OR = 1.41, 95%CI 0.90 to 2.20), but no longer reached conventional levels of significance. For males, the effect estimate was similar to that found for females, however the confidence interval was wide (fully adjusted OR = 1.51, 95%CI 0.73 to 3.14). We did not find evidence to suggest an association between the level of internet use and anxiety for either gender. With regards to self-harm, we detected an association between level of internet use and an increased odds of self-harm for males but not for females (Table 1); In fully adjusted models, the odds of self-harm were 2.5 times higher for males in the highest tertile compared to those in the lowest tertile (OR = 2.53, 95%CI 0.93 to 6.90).

**Table 1:** Association between internet use and mental health outcomes: Imputed data.

	Proportion reporting outcome	OR (95%CI) Unadjusted	OR (95% CI) Adjusted for SEP	OR (95% CI) Additionally adjusted for previous mental health problems
**Depression**				
**Tertiles: Males (n = 536)**				
0–12 (n = 200)	14.6%	1.00	1.00	1.00
13–21 (n = 164)	15.3%	1.07 (0.54, 2.11)	1.13 (0.57, 2.27)	1.28 (0.61, 2.69)
22–132 (n = 172)	19.2%	1.40 (0.71, 2.74)	1.46 (0.74, 2.91)	1.51 (0.73, 3.14)
**Tertiles: Females (n = 895)**				
1–8 (n = 333)	19.5%	1.00	1.00	1.00
9–15 (n = 284)	17.6%	0.88 (0.56, 1.37)	0.87 (0.56, 1.36)	0.85 (0.53, 1.36)
16–72 (n = 278)	27.9%	1.60 (1.06, 2.44)	1.61 (1.06, 2.45)	1.41 (0.90, 2.20)
**Anxiety**				
**Tertiles: Males (n = 536)**				
0–12 (n = 200)	8.9%	1.00	1.00	1.00
13–21 (n = 164)	9.2%	1.04 (0.46, 2.36)	1.07 (0.47, 2.46)	1.40 (0.53, 3.70)
22–132 (n = 172)	9.4%	1.07 (0.43, 2.63)	1.15 (0.46, 2.88)	1.22 (0.43, 3.47)
**Tertiles: Females (n = 895)**				
1–8 (n = 333)	12.4%	1.00	1.00	1.00
9–15 (n = 284)	12.4%	1.00 (0.57, 1.75)	0.98 (0.56, 1.71)	0.95 (0.53, 1.72)
16–72 (n = 278)	17.5%	1.49 (0.88, 2.52)	1.48 (0.88, 2.49)	1.28 (0.74, 2.22)
**Self-harm**				
**Tertiles: Males (n = 536)**				
0–12 (n = 200)	7.1%	1.00	1.00	1.00
13–21 (n = 164)	9.0%	1.29 (0.45, 3.66)	1.30 (0.45, 3.73)	1.30 (0.45, 3.79)
22–132 (n = 172)	14.4%	2.26 (0.84, 6.07)	2.52 (0.92, 6.87)	2.53 (0.93, 6.90)
**Tertiles: Females (n = 895)**				
1–8 (n = 333)	12.6%	1.00	1.00	1.00
9–15 (n = 284)	11.8%	0.92 (0.52, 1.63)	0.91 (0.51, 1.62)	0.87 (0.48, 1.60)
16–72 (n = 278)	15.1%	1.24 (0.72, 2.11)	1.22 (0.71, 2.09)	1.05 (0.61, 1.83)

### Internet experiences

**Fig 2:**
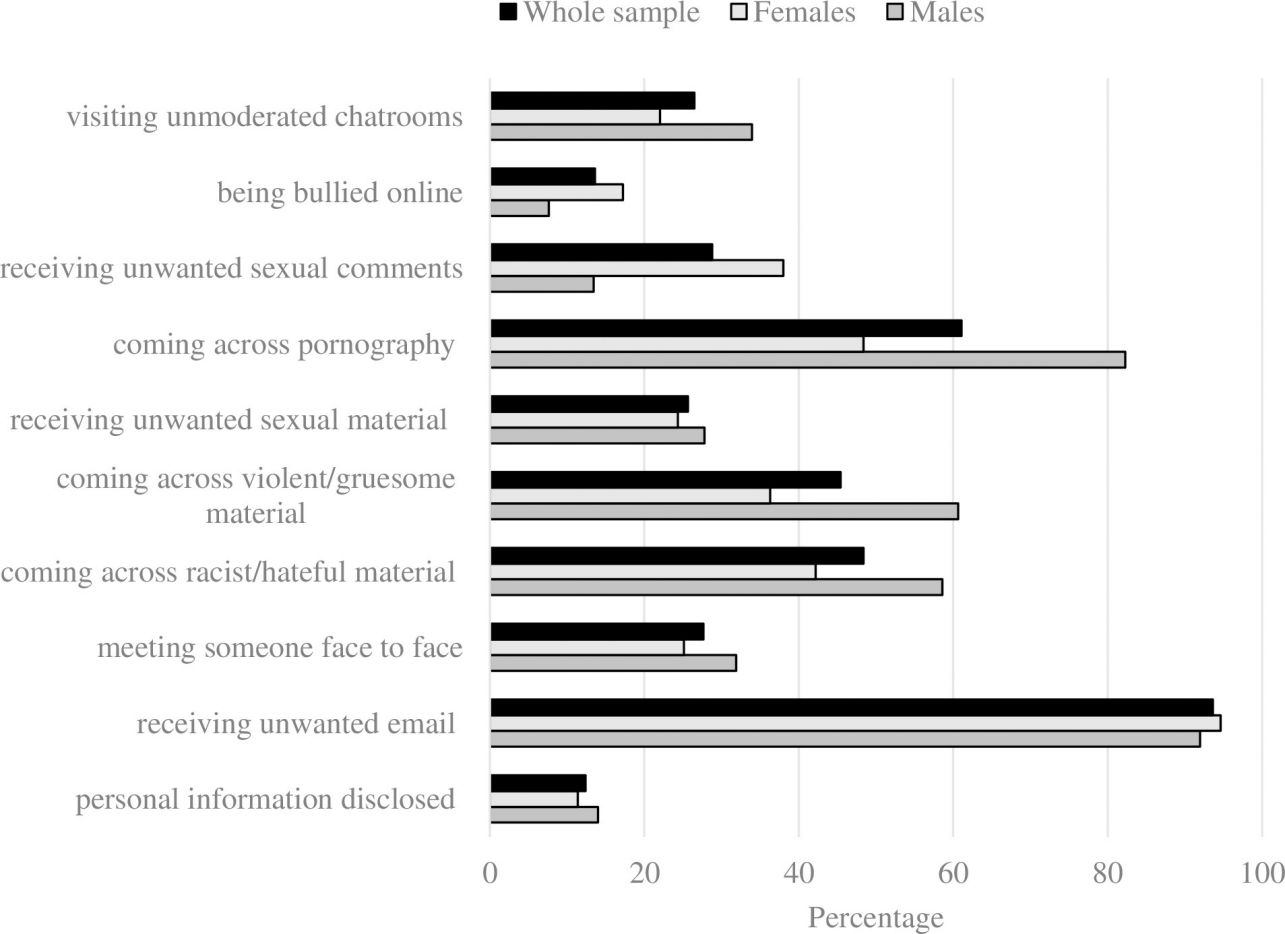
Proportion of the sample reporting internet experiences.

The proportion of the sample endorsing each of the ten different scenarios is displayed in Fig 2. The prevalence ranged from 12.4% (having personal information disclosed) to 93.6% (receiving spam/unwanted email). The prevalence of visiting unmoderated chatrooms, meeting someone face-to-face and coming across pornography, violent/gruesome material, and racist/hateful material was higher in males. Females had a higher prevalence of being bullied and receiving unwanted sexual comments. The proportion receiving unwanted sexual material, receiving unwanted email and having personal information disclosed was similar across genders.

#### Depression

Associations between each of the ten internet experiences and the odds of later depression are shown in Table 2. There was little evidence to suggest an association between any of the internet experiences and the odds of later depression for males. For females, associations were found in unadjusted models for seven out of the ten online experiences, however after adjustment for confounders (SEP, hours online and earlier mental health problems), associations remained only for being bullied (OR = 1.76. 95%CI 1.09 to 2.86) and meeting someone face to face (OR = 1.55, 95%CI 1.00 to 2.41).

**Table 2:** Association between online experiences and high risk of depression: Imputed data.

	OR (95% CI) Unadjusted	OR (95% CI) Adjusted for SEP and hours online	OR (95% CI) Additionally adjusted for previous mental health problems
**Unmoderated chatrooms**			
Males	1.62 (0.93, 2.80)	1.49 (0.84, 2.67)	1.38 (0.76, 2.51)
Females	1.56 (0.99, 2.47)	1.43 (0.91, 2.26)	1.29 (0.79, 2.09)
**Being bullied online**			
Males	1.75 (0.61, 5.01)	1.50 (0.49, 4.56)	1.28 (0.39, 4.19)
Females	2.24 (1.43, 3.51)	2.22 (1.41, 3.49)	1.76 (1.09, 2.86)
**Unwanted sexual comments**			
Males	1.94 (0.87, 4.31)	1.65 (0.72, 3.81)	1.50 (0.62, 3.77)
Females	1.32 (0.93, 1.88)	1.28 (0.89, 1.82)	1.03 (0.70, 1.52)
**Coming across pornography**			
Males	0.98 (0.46, 2.05)	0.95 (0.45, 2.02)	0.88 (0.40, 1.97)
Females	1.69 (1.16, 2.45)	1.60 (1.10, 2.33)	1.33 (0.89, 1.99)
**Unwanted sexual material**			
Males	0.91 (0.47, 1.76)	0.89 (0.45, 1,76)	0.86 (0.42, 1,75)
Females	1.77 (1.18, 2.64)	1.72 (1.15, 2.58)	1.38 (0.89, 2.13)
**Violent/gruesome material**			
Males	1.44 (0.76, 2.71)	1.32 (0.68, 2.56)	1.21 (0.60, 2.42)
Females	1.57 (1.10, 2.23)	1.51 (1.05, 2.17)	1.21 (0.81, 1.78)
**Racist/hateful material**			
Males	1.42 (0.77, 2.60)	1.40 (0.74, 2.62)	1.23 (0.64, 2.39)
Females	1.48 (1.02, 2.14)	1.42 (0.97, 2.08)	1.15 (0.76, 1.74)
**Meeting face to face**			
Males	1.68 (0.90, 3.13)	1.44 (0.76, 2.72)	1.30 (0.67, 2.53)
Females	2.03 (1.37, 3.02)	1.98 (1.31, 2.98)	1.55 (1.00, 2.41)
**Personal information disclosed**			
Males	1.47 (0.71, 3.01)	1.44 (0.69, 2.98)	1.34 (0.63, 2.86)
Females	1.58 (0.84, 2.97)	1.53 (0.80, 2.91)	1.18 (0.59, 2.35)
**Junk mail /unwanted email**			
Males	0.52 (0.22, 1.24)	0.45 (0.18, 1.12)	0.47 (0.17, 1.28)
Females	1.13 (0.45, 2.85)	1.07 (0.42, 2.70)	0.70 (0.26, 1.87)

#### Anxiety

Associations between each of the ten internet experiences and later anxiety symptoms are shown in Table 3. In unadjusted models, visiting unmoderated chatrooms, being bullied and receiving unwanted sexual comments were associated with an increased risk of anxiety for both males and females. For females, associations were also found for receiving unwanted sexual material and for coming across violent/gruesome and racist/hateful material. However, all findings attenuated to the null following adjustment for confounders.

**Table 3:** Association between online experiences and high risk of anxiety disorder: Imputed data.

	OR (95% CI) Unadjusted	OR (95% CI) Adjusted for SEP and hours online	OR (95% CI) Additionally adjusted for previous mental health problems
**Unmoderated chatrooms**			
Males	2.48 (1.06, 5.80)	2.46 (1.02, 5.95)	2.35 (0.87, 6.33)
Females	1.65 (0.98, 2.77)	1.53 (0.90, 2.58)	1.37 (0.78, 2.39)
**Being bullied online**			
Males	3.35 (1.13, 9.95)	3.02 (0.95, 9.58)	2.59 (0.66, 10.1)
Females	1.87 (1.14, 3.08)	1.83 (1.11, 3.04)	1.40 (0.81, 2.40)
**Unwanted sexual comments**			
Males	2.53 (1.01, 6.37)	2.37 (0.91, 6.16)	2.26 (0.77, 6.62)
Females	1.51 (0.97, 2.36)	1.46 (0.93, 2.29)	1.18 (0.73, 1.91)
**Coming across pornography**			
Males	1.00 (0.37, 2.69)	0.98 (0.35, 2.76)	0.92 (0.28, 2.99)
Females	1.38 (0.88, 2.17)	1.31 (0.84, 2.06)	1.05 (0.65, 1.70)
**Unwanted sexual material**			
Males	0.73 (0.31, 1.72)	0.72 (0.30, 1.71)	0.66 (0.26, 1.71)
Females	1.63 (1.02, 2.61)	1.61 (1.00, 2.61)	1.26 (0.75, 2.13)
**Violent/gruesome material**			
Males	1.79 (0.81, 3.95)	1.75 (0.75, 4.10)	1.61 (0.63, 4.10)
Females	1.80 (1.16, 2.78)	1.77 (1.13, 2.77)	1.41 (0.88, 2.26)
**Racist/hateful material**			
Males	1.26 (0.60, 2.66)	1.23 (0.54, 2.83)	1.02 (0.42, 2.44)
Females	1.54 (1.01, 2.34)	1.51 (0.97, 2.34)	1.22 (0.75, 1.97)
**Meeting face to face**			
Males	1.69 (0.81, 3.51)	1.59 (0.73, 3.42)	1.40 (0.59, 3.28)
Females	1.50 (0.93, 2.43)	1.42 (0.86, 2.33)	1.05 (0.61, 1.81)
**Personal information disclosed**			
Males	1.25 (0.42, 3.75)	1.28 (0.40, 4.10)	1.13 (0.33, 3.84)
Females	1.34 (0.72, 2.50)	1.29 (0.68, 2.43)	0.95 (0.49, 1.88)
**Junk mail /unwanted email**			
Males	0.50 (0.16, 1.55)	0.40 (0.12, 1.31)	0.45 (0.11, 1.86)
Females	1.05 (0.39, 2.81)	1.00 (0.37, 2.67)	0.61 (0.22, 1.77)

#### Self-harm

Associations between each of the ten internet experiences and past year self-harm are displayed in Table 4. For males, only meeting someone face to face was associated with an increased risk of self-harm (OR = 2.38, 95%CI 1.03 to 5.52), however the effect did not remain after adjustment for confounders (OR = 2.18, 95%CI 0.89 to 5.32). For females, the overall pattern of results was similar across unadjusted and adjusted models. In fully adjusted models, associations were found for being bullied online (OR = 2.42, 95%CI 1.41 to 4.15), receiving unwanted sexual comments (OR = 1.88, 95%CI 1.14 to 3.08), coming across pornography (OR = 2.14, 95%CI 1.28 to 3.58), receiving unwanted sexual material (OR = 1.95, 95%CI 1.20 to 3.16), and also coming across violent/gruesome material (OR = 1.78, 95%CI 1.11 to 2.85). We did not find evidence for an association with coming across racist/hateful material or with meeting someone face to face following adjustment for confounders.

**Table 4:** Association between online experiences and past year self-harm: Imputed data.

	OR (95% CI) Unadjusted	OR (95% CI) Adjusted for SEP and hours online	OR (95% CI) Additionally adjusted for previous mental health problems
**Unmoderated chatrooms**			
Males	1.56 (0.68, 3.54)	1.29 (0.52, 3.20)	1.30 (0.53, 3.20)
Females	1.32 (0.81, 2.15)	1.23 (0.74, 2.04)	1.09 (0.65, 1.84)
**Being bullied online**			
Males	1.75 (0.39, 7.77)	1.55 (0.35, 6.93)	1.59 (0.35, 7.26)
Females	3.01 (1.82, 4.96)	2.96 (1.78, 4.92)	2.42 (1.41, 4.15)
**Unwanted sexual comments**			
Males	1.20 (0.41, 3.56)	1.03 (0.33, 3.28)	1.03 (0.33, 3.27)
Females	2.27 (1.44, 3.58)	2.19 (1.38, 3.48)	1.88 (1.14, 3.08)
**Coming across pornography**			
Males	1.46 (0.40, 5.34)	1.20 (0.31, 4.60)	1.21 (0.32, 4.60)
Females	2.48 (1.52, 4.06)	2.47 (1.50, 4.06)	2.14 (1.28, 3.58)
**Unwanted sexual material**			
Males	1.00 (0.46, 2.14)	0.96 (0.43, 2.13)	0.96 (0.43, 2.13)
Females	2.34 (1.48, 3.70)	2.35 (1.48, 3.70)	1.95 (1.20, 3.16)
**Violent/gruesome material**			
Males	1.26 (0.62, 2.54)	1.05 (0.50, 2.20)	1.05 (0.49, 3.39)
Females	2.15 (1.37, 3.39)	2.14 (1.36, 3.72)	1.78 (1.11, 2.85)
**Racist/hateful material**			
Males	1.10 (0.51, 2.37)	0.91 (0.41, 2.02)	0.91 (0.41, 2.05)
Females	1.64 (1.06, 2.55)	1.65 (1.06, 2.57)	1.38 (0.86, 2.20)
**Meeting face to face**			
Males	2.38 (1.03, 5.52)	2.16 (0.90, 5.20)	2.18 (0.89, 5.32)
Females	2.00 (1.21, 3.32)	1.89 (1.14, 3.13)	1.51 (0.88, 2.58)
**Personal information disclosed**			
Males	0.82 (0.28, 2.38)	0.81 (0.27, 2.43)	0.81 (0.72, 2.91)
Females	1.45 (0.71, 2.91)	1.41 (0.69, 2.88)	1.10 (0.51, 2.37)
**Junk mail /unwanted email**			
Males	1.01 (0.22, 4.73)	0.72 (0.14, 3.65)	0.71 (0.14, 3.66)
Females	3.02 (0.70, 12.9)	2.95 (0.68,12.8)	2.05 (0.45, 9.26)

#### Secondary analysis: Total number of experiences

With the exception of receiving unwanted email and visiting unmoderated chatrooms, each of the internet experiences was strongly correlated with one another (S4 Table). Table 5 shows associations between the total number of internet experiences (range 0–10) and mental health outcomes. The distribution of experiences across the sample is provided in S5 Table.

**Table 5:** Association between total number of online experiences and mental health outcomes: Imputed data.

	OR (95%CI) Unadjusted	OR (95% CI) Adjusted for SEP	OR (95% CI) Additionally adjusted for previous mental health problems
**Depression**			
**Males**	1.12 (0.97, 1.30)	1.09 (0.93, 1.28)	1.06 (0.90, 1.24)
**Females**	1.18 (1.09, 1.27)	1.17 (1.07, 1.27)	1.09 (0.99, 1.20)
**Anxiety**			
**Males**	1.17 (0.97, 1.41)	1.15 (0.95, 1.42)	1.11 (0.90, 1.37)
**Females**	1.16 (1.06, 1.26)	1.15 (1.05, 1.26)	1.06 (0.96, 1.19)
**Self-harm**			
**Males**	1.12 (0.93, 1.34)	1.06 (0.87, 1.30)	1.07 (0.87, 1.31)
**Females**	1.27 (1.16, 1.38)	1.27 (1.16, 1.39)	1.20 (1.09, 1.33)

For females, the total number of internet experiences was associated with an increased risk of depression (OR per additional experience = 1.09, 95%CI 0.99, 1.20), anxiety (OR per additional experience = 1.06, 95%CI 0.96, 1.19) and self-harm (OR per additional experience = 1.20, 95% CI 1.09, 1.33) (although findings for depression and anxiety did not reach conventional levels of significance). There was little evidence for an association in males.

## Discussion

In this longitudinal study, we found that females reporting high levels of internet use were at increased risk of future depression and males were at increased risk for self-harm. Risk of adverse outcomes were also increased for females following exposure to specific online experiences, most notably being bullied online, meeting someone face to face, receiving unwanted sexual comments/material, and coming across pornography or violent/gruesome material. Risk of adverse outcomes was also increased for each additional experience reported. Associations remained following adjustment for earlier mental health problems, suggesting that findings were not explained by pre-existing levels of psychopathology. In contrast to depression and self-harm, internet use was not associated with anxiety (in fully adjusted models). Additional longitudinal studies are needed to see if our findings are replicated. Further research is also needed to explore the mechanisms through which internet use impacts on mental health to better understand any potential differences in outcome.

Strengths of the study include the longitudinal design, adjustment for confounders, and exploration of potential gender differences. However, findings should be interpreted in light of several limitations. Firstly, the questions used to assess Internet use were developed for the ALSPAC study and have not previously been validated. The measures used were also self-reported and participants may not have recalled this information accurately. Moreover, ‘hours online’ is a simplistic measure, and it is likely that associations between internet use and mental health are more complex, for example associations may differ for active vs passive use,[34] according to the type of activity (e.g. social networking, gaming etc.), or according to frequency of exposure (the low numbers in some categories precluded our ability to look at frequency). We were also unable to distinguish between intentional and unintentional exposure to potentially harmful material. Second, we explored associations in a large sample of nearly 1,500 individuals, however, the level of response to the questionnaire was low (16%). This is likely due to the lack of reminders and to the unfamiliar format of the questionnaire, which was the first in ALSPAC to be administered online. There were some differences found between those who did and did not return the questionnaire, which may affect the generalisability of the results. Third, data on self-harm, depression and anxiety were not collected at the same time point and its possible this may have affected our estimates (i.e. if stronger associations are found for data collected closer in time). Finally, since the time of data collection (2010) the growth in ownership of smartphones[1] has changed the way in which the internet is accessed and used. The average number of hours spent online per week is lower in this study than found in a recent survey (14 hours vs 21 hours).[1] However, online experiences such as cyberbullying are still very relevant issues and the increased time spent online may serve to increase the prevalence and impact of these exposures.

Previous research has been limited by a reliance on cross-sectional data and it is currently unclear whether high-levels of internet use are a correlate, consequence or risk factor for psychopathology. We provide an important extension to previous work by exploring relationships over time and have shown that high levels of internet use are prospectively associated with an increased risk of future depression for females and self-harm for males, even after adjusting for existing mental health symptoms. This gender difference in outcome could be due to differences in types of Internet use by males and females.[19] We found males were more likely than females to report exposure to pornography, and violent/gruesome content (see Fig 2). However, males who reported these experiences were not found to be at increased risk of self-harm in this study.

Although some prior studies have explored the relationship between specific online experiences and mental health, including pornography and chatrooms,[8, 35–37] longitudinal research is scarce. Cyberbullying has received the most attention in the literature [17] and has been found to be related to a wide range of adverse outcomes, including suicidal ideation, depression, anxiety and self-harm.[4, 5] Consistent with our findings, the few previous longitudinal investigations have found that being bullied online has a detrimental effect on later mental health, with some studies finding evidence of a reciprocal relationship.[6, 7, 38–41] However, previous research has focused predominantly on adolescents, and to our knowledge, this is the first longitudinal study to explore outcomes of cyberbullying in early adulthood. In our study, data was only available on cybervictimization, but future research should also investigate associations among cyberbullying perpetrators.

Our findings emphasise the importance of digital citizenship training from an early age and suggest that services supporting young people with mental health issues should ask about their Internet use. Regulation is also needed to establish online safety measures that help counter potentially harmful activity or content, whilst maximising the positive aspects of Internet use.[42]. Future research in this area should consider the role of gender in more detail and use prospective designs to establish the direction of effects between internet use and adverse mental health, as well as exploring potential mechanisms underlying associations. Qualitative studies will also provide useful insight into how online content is used and the impact of context and content on mental health.[43]

## Supporting information

S1 Table Comparison of responders and non-responders to the internet use questionnaire at 18 years by key demographic variables (comparison amongst those invited to participate in the questionnaire, N = 9,160). (DOCX)

S2 Table Association between internet use (hours online) and mental health outcomes—comparison of complete case and imputed data. (DOCX)

S3 Table Association between internet experiences and mental health outcomes—comparison of complete case and imputed data. (DOCX)

S4 Table Correlations between internet experiences. (DOCX)

S5 Table Distribution of the total number of internet experiences reported. (DOCX)
