# Supplementary material for: Prospective associations between internet use and poor mental health: A population-based study
Source: PLoS One. 2020 Jul 23;15(7):e0235889. doi: 10.1371/journal.pone.0235889 (PMC7377422; doi:10.1371/journal.pone.0235889)
Supplement: S1 Table — (DOCX) [file pone.0235889.s001.docx]

**S1 Table. Comparison of responders and non-responders to the internet use questionnaire at 18 years by key demographic variables (Comparison amongst those invited to participate in the questionnaire, *N*=9,160)**

| Variable | Description | *N* | No data on internet use ^a^ | Complete data on internet use | χ^2^ | *P* value |
| --- | --- | --- | --- | --- | --- | --- |
| Child gender | Male | 4,474 | 3,938 (51.0%) | 536 (37.5%) | 88.0 | <0.001 |
|  | Female | 4,686 | 3,791 (49.1%) | 895(62.5%) |  |  |
| Parity | First born | 3,993 | 3,288 (44.7%) | 705 (50.4%) | 18.3 | <0.001 |
|  | Second born | 3,115 | 2,640 (35.9%) | 475 (34.0%) |  |  |
|  | Third born plus | 1,645 | 1,426 (19.4%) | 219 (15.6%) |  |  |
| Home overcrowding | <1 person/room | 8,235 | 6,891 (94.4%) | 1,344 (96.9%) | 14.6 | <0.001 |
|  | >1 person/room | 450 | 407 (5.6%) | 43 (3.1%) |  |  |
| Maternal education | Degree | 1,289 | 950 (13.1%) | 339 (24.3%) | 174.7 | <0.001 |
|  | A level | 2,162 | 1,756 (24.2%) | 406 (29.0%) |  |  |
|  | O level | 3,029 | 2,598 (35.8%) | 431 (30.8%) |  |  |
|  | < O level | 2,180 | 1,958 (26.9%) | 222 (15.9%) |  |  |
| Parental social class | Professional/managerial | 4,761 | 3,856 (56.5%) | 905 (67.4%) | 55.2 | <0.001 |
|  | other | 3,413 | 2,975 (43.5%) | 438 (32.6%) |  |  |
| Child ethnicity | White | 8,107 | 6,785 (95.4%) | 1,322 (96.0%) | 1.1 | 0.284 |
|  | Non-white | 386 | 331 (4.6%) | 55 (4.0%) |  |  |

*^a^ Includes those who were sent but did not return the internet use questionnaire, and those who returned the questionnaire but did not have complete data on the exposures of interest*
