# Supplementary material for: Prospective associations between internet use and poor mental health: A population-based study
Source: PLoS One. 2020 Jul 23;15(7):e0235889. doi: 10.1371/journal.pone.0235889 (PMC7377422; doi:10.1371/journal.pone.0235889)
Supplement: S2 Table — (DOCX) [file pone.0235889.s002.docx]

**S2 Table. Association between internet use (hours online) and mental health outcomes - comparison of complete case and imputed data**

| **Hours spent online** | **Depression** | | **Anxiety** | | **Self-harm** | |
| --- | --- | --- | --- | --- | --- | --- |
|  | **Imputed data** | **Complete case** | **Imputed data** | **Complete case** | **Imputed data** | **Complete case** |
| **Tertiles: Males**  0-12  13-21  22-132 | 1.00  1.28 (0.61, 2.69)  1.51 (0.73, 3.14) | 1.00  1.85 (0.65, 5.25)  1.43 (0.49, 4.22) | 1.00  1.40 (0.53, 3.70)  1.22 (0.43, 3.47) | 1.00  4.31 (0.98, 18.9)  0.47 (0.07, 3.27) | 1.00  1.30 (0.45, 3.79)  2.53 (0.93, 6.90) | 1.00  0.37 (0.09, 1.53)  * |
| **Tertiles: Females**  1-8  9-15  16-72 | 1.00  0.85 (0.53, 1.36)  1.41 (0.90, 2.20) | 1.00  0.83 (0.47, 1.46)  1.61 (0.95, 2.72) | 1.00  0.95 (0.53, 1.72)  1.28 (0.74, 2.22) | 1.00  0.93 (0.49, 1.78)  1.25 (0.67, 2.35) | 1.00  0.87 (0.48, 1.60)  1.05 (0.61, 1.83) | 1.00  0.73 (0.35, 1.51)  1.19 (0.61, 2.30) |

*Models adjusted for maternal education, social class, and previous depression symptoms*

*Imputed sample N=1,431. Number with missing data = 33 for maternal education; 88 for social class; 261 for depressive symptoms; 383 for self-harm; 469 for anxiety=469 and 481 for depression.*

** The association could not be calculated due to small numbers*
