# Supplementary material for: Prospective associations between internet use and poor mental health: A population-based study
Source: PLoS One. 2020 Jul 23;15(7):e0235889. doi: 10.1371/journal.pone.0235889 (PMC7377422; doi:10.1371/journal.pone.0235889)
Supplement: S3 Table — (DOCX) [file pone.0235889.s003.docx]

**S3 Table. Association between internet experiences and mental health outcomes - comparison of complete case and imputed data**

| **Internet experiences** | **Depression** | | **Anxiety** | | **Self-harm** | |
| --- | --- | --- | --- | --- | --- | --- |
|  | **Imputed data** | **Complete case** | **Imputed data** | **Complete case** | **Imputed data** | **Complete case** |
| **Unmoderated chatrooms**  Males | 1.38 (0.76, 2.51) | 1.38 (0.55, 3.41) | 2.35 (0.87, 6.33) | 3.48 (1.02, 11.8) | 1.30 (0.53, 3.20) | 1.82 (0.44, 7.48) |
| Females | 1.29 (0.79, 2.09) | 1.39 (0.84, 2.30) | 1.37 (0.78, 2.39) | 1.42 (0.79, 2.54) | 1.09 (0.65, 1.84) | 0.72 (0.35, 1.48) |
| **Being bullied online**  Males | 1.28 (0.39, 4.19) | 0.57 (0.06, 5.72) | 2.59 (0.66, 10.1) | 0.91 (0.09, 9.03) | 1.59 (0.35, 7.26) | 1.52 (0.15, 15.4) |
| Females | 1.76 (1.09, 2.86) | 1.86 (1.07, 3.23) | 1.40 (0.81, 2.40) | 1.41 (0.73, 2.73) | 2.42 (1.41, 4.15) | 2.40 (1.26, 4.60) |
| **Unwanted sexual comments**  Males | 1.50 (0.62, 3.77) | 1.33 (0.41, 4.27) | 2.26 (0.77, 6.62) | 1.35 (0.30, 6.00) | 1.03 (0.33, 3.27) | 1.85 (0.31, 10.9) |
| Females | 1.03 (0.70, 1.52) | 0.87 (0.54, 1.38) | 1.18 (0.73, 1.91) | 1.04 (0.60, 1.78) | 1.88 (1.14, 3.08) | 1.66 (0.94, 2.93) |
| **Coming across pornography**  Males | 0.88 (0.40, 1.97) | 1.78 (0.46, 6.85) | 0.92 (0.28, 2.99) | 1.06 (0.19, 6.04) | 1.21 (0.32, 4.60) | * |
| Females | 1.33 (0.89, 1.99) | 1.63 (1.04, 2.57) | 1.05 (0.65, 1.70) | 1.07 (0.63, 1.81) | 2.14 (1.28, 3.58) | 2.23 (1.23, 4.04) |
| **Unwanted sexual material**  Males | 0.86 (0.42, 1,75) | 0.81 (0.30, 2.22) | 0.66 (0.26, 1.71) | 0.60 (0.15, 2.35) | 0.96 (0.43, 2.13) | 0.88 (0.17, 4.49) |
| Females | 1.38 (0.89, 2.13) | 1.42 (0.87, 2.32) | 1.26 (0.75, 2.13) | 1.19 (0.66, 2.13) | 1.95 (1.20, 3.16) | 1.78 (0.96, 3.30) |
| **Violent/gruesome material**  Males | 1.21 (0.60, 2.42) | 1.69 (0.65, 4.38) | 1.61 (0.63, 4.10) | 3.78 (0.83, 17.2) | 1.05 (0.49, 3.39) | 2.17 (0.41, 11.4) |
| Females | 1.21 (0.81, 1.78) | 1.18 (0.74, 1.87) | 1.41 (0.88, 2.26) | 1.48 (0.87, 2.53) | 1.78 (1.11, 2.85) | 1.68 (0.94, 3.00) |
| **Racist/hateful material**  Males | 1.23 (0.64, 2.39) | 1.45 (0.57, 3.69) | 1.02 (0.42, 2.44) | 0.99 (0.29, 3.38) | 0.91 (0.41, 2.05) | 1.17 (0.26, 5.26) |
| Females | 1.15 (0.76, 1.74) | 1.11 (0.70, 1.76) | 1.22 (0.75, 1.97) | 1.18 (0.69, 2.02) | 1.38 (0.86, 2.20) | 1.11 (0.62, 2.00) |
| **Meeting face to face**  Males | 1.30 (0.67, 2.53) | 0.83 (0.32, 2.19) | 1.40 (0.59, 3.28) | 0.38 (0.08, 1.72) | 2.18 (0.89, 5.32) | 5.20 (1.20, 22.5) |
| Females | 1.55 (1.00, 2.41) | 1.85 (1.14, 2.99) | 1.05 (0.61, 1.81) | 0.97 (0.53, 1.77) | 1.51 (0.88, 2.58) | 1.65 (0.90, 3.01) |
| **Personal information disclosed**  Males | 1.34 (0.63, 2.86) | 1.44 (0.43, 4.82) | 1.13 (0.33, 3.84) | 0.97 (0.17, 5.47) | 0.81 (0.72, 2.91) | 0.75 (0.08, 6.66) |
| Females | 1.18 (0.59, 2.35) | 1.13 (0.56, 2.28) | 0.95 (0.49, 1.88) | 0.87 (0.36, 2.06) | 1.10 (0.51, 2.37) | 0.80 (0.32, 2.05) |
| **Junk mail /unwanted email**  Males | 0.47 (0.17, 1.28) | 0.40 (0.10, 1.65) | 0.45 (0.11, 1.86) | 0.22 (0.04, 1.26) | 0.71 (0.14, 3.66) | 0.46 (0.05, 4.26) |
| Females | 0.70 (0.26, 1.87) | 0.45 (0.14, 1.52) | 0.61 (0.22, 1.77) | 0.77 (0.16, 3.64) | 2.05 (0.45, 9.26) | 1.75 (0.22, 13.6) |

*Models adjusted for maternal education, social class, previous depression symptoms and hours spent online*

*Imputed sample N=1,431. Number with missing data = 33 for maternal education; 88 for social class; 261 for depressive symptoms; 383 for self-harm; 469 for anxiety=469 and 481 for depression.*

** The association could not be calculated due to small numbers*
