# Supplementary material for: Prospective associations between internet use and poor mental health: A population-based study
Source: PLoS One. 2020 Jul 23;15(7):e0235889. doi: 10.1371/journal.pone.0235889 (PMC7377422; doi:10.1371/journal.pone.0235889)
Supplement: S4 Table — (DOCX) [file pone.0235889.s004.docx]

**S4 Table. Correlations between Internet experiences**

|  | Chatrooms | Cyberbullied | Sexual comments | Pornography | Sexual material | Violent/gruesome material | Racist/hateful material | Meeting face to face | Personal info | Unwanted email |
| --- | --- | --- | --- | --- | --- | --- | --- | --- | --- | --- |
| Chatrooms | 1 |  |  |  |  |  |  |  |  |  |
| Cyberbullied | 0.168** | 1 |  |  |  |  |  |  |  |  |
| Sexual comments | 0.257** | 0.512** | 1 |  |  |  |  |  |  |  |
| Pornography | 0.411** | 0.187** | 0.349** | 1 |  |  |  |  |  |  |
| Sexual material | 0.352** | 0.301** | 0.536** | 0.599** | 1 |  |  |  |  |  |
| Violent/gruesome material | 0.426** | 0.287** | 0.250** | 0.695** | 0.550** | 1 |  |  |  |  |
| Racist/hateful material | 0.375** | 0.306** | 0.276** | 0.635** | 0.471** | 0.808** | 1 |  |  |  |
| Meeting face to face | 0.253** | 0.317** | 0.294** | 0.268** | 0.219** | 0.265** | 0.266** | 1 |  |  |
| Personal info | 0.267** | 0.219** | 0.374** | 0.372** | 0.541** | 0.422** | 0.471** | 0.355** | 1 |  |
| Unwanted email | 0.244** | 0.082** | 0.398** | 0.419** | 0.459** | 0.462** | 0.437** | 0.1816* | 0.222* | 1 |

*Correlations are for complete case data*
