# Supplementary material for: Prospective associations between internet use and poor mental health: A population-based study
Source: PLoS One. 2020 Jul 23;15(7):e0235889. doi: 10.1371/journal.pone.0235889 (PMC7377422; doi:10.1371/journal.pone.0235889)
Supplement: S5 Table — (DOCX) [file pone.0235889.s005.docx]

**S5 Table. Distribution of the total number of Internet experiences reported**

| Number of experiences reported | Proportion of total sample | Males | Females |
| --- | --- | --- | --- |
| 0 | 3.6 | 3.5 | 3.6 |
| 1 | 14.3 | 6.2 | 19.2 |
| 2 | 14.1 | 11.8 | 15.5 |
| 3 | 16.1 | 16.8 | 15.6 |
| 4 | 15.4 | 16.2 | 15.0 |
| 5 | 12.7 | 18.5 | 9.3 |
| 6 | 9.6 | 14.2 | 6.9 |
| 7 | 7.1 | 6.2 | 7.6 |
| 8 | 4.6 | 4.5 | 4.7 |
| 9 | 1.7 | 1.5 | 1.8 |
| 10 | 0.8 | 0.8 | 0.8 |
